# Preliminary experience in laparoscopic distal pancreatectomy using the AEON™ endovascular stapler

**DOI:** 10.3389/fonc.2023.1146646

**Published:** 2023-04-12

**Authors:** Aali J. Sheen, Samik Bandyopadhyay, Minas Baltatzis, Rahul Deshpande, Saurabh Jamdar, Nicola de Liguori Carino

**Affiliations:** ^1^ Manchester University Foundation National Health Service (NHS) Trust, Department of Surgery, Manchester, United Kingdom; ^2^ Faculty of Biology, Medicine and Health (FBMH), University of Manchester, Manchester, United Kingdom; ^3^ Faculty of Bioscience, Manchester Metropolitan University, Manchester, United Kingdom; ^4^ Department of General Surgery, Salford Royal Foundation Trust, Salford, United Kingdom

**Keywords:** distal pancreatectomy, pancreatic fistula, endovascular stapler, AEON stapler, drain lipase

## Abstract

**Background:**

The aim of this study is to investigate the effects of using a new innovative endovascular stapler, AEON™, on the pancreatic leak rates and other outcome measures.

**Methods:**

In a retrospective review of prospectively collected data from a secure tertiary unit registry, patients undergoing distal or lateral pancreatectomy were analyzed for any differences on pancreatic fistula rates, length of stay, comprehensive complication index (CCI), and demographics after using AEON™ compared with other commonly used staplers. Statistical significance was defined as <0.05.

**Results:**

There were no differences in the demographics between the two groups totaling 58 patients over 2 years from 2019 to 2021. A total of 43 and 15 patients underwent pancreatic transection using other staplers and AEON™ endovascular stapler, respectively. The comparison of the two groups revealed a significantly reduced rate of mean drain lipase at postoperative day 3 with AEON™ (446 U/L) versus the other staplers (4,208 U/L) (p = 0.018) and a subsequent reduction of postoperative pancreatic fistula (POPF) from 65% to 20%. A reduction in the mean CCI, from 13.80 when other staplers were used to 4.97 when AEON™ was used, was also observed (p = 0.087). Mean length of stay was shorter by 3 days in the AEON™ group compared with that in the other staplers (6 and 9 days, respectively; p = 0.018).

**Conclusion:**

AEON™ stapler when used to transect the pancreas demonstrated a significantly reduced pancreatic fistula rate, length of stay in hospital, and a leaning towards a reduced CCI. Its use should be further evaluated in larger cohorts with the encouraging results to determine whether this is possibly related to the technology used in the design of the AEON™ stapler.

## Introduction

Laparoscopic surgery on the pancreas particularly for distal pancreatectomy and for small enucleations has evolved since the mid-1990s (1). Initially with laparoscopic surgery, only benign tumors were operated because of the concerns regarding safety and oncological clearance (2), but, now, surgery using the minimally invasive route has become the normal practice even in low resource countries (3). Laparoscopic surgery has provided many advantages such as the improved visualization of the pancreas, the splenic vessels, and other surrounding structures as well as the improved magnification aiding in surgical dissection (4, 5). However, the main caveat remains that it still is a high morbidity procedure with iatrogenic life-threatening bleeding that is always a possibility, but, increasingly, the laparoscopic technique has shown an overall reduced blood loss with less pain. However, the pancreatic leak rates have remained the same (6). Importantly, it has now become such a routine procedure that there are comparisons being made with innovative minimal access techniques such as robotic surgery, with, again, no difference in the pancreatic fistula rates (24.6% versus 26.5%; P = 0.543) (7). Pancreatic fistula rates can vary between 20% and 40% or sometimes higher as described by many studies over the years; grade B/C postoperative pancreatic fistulas (POPFs) were seen in 39% after minimally invasive distal pancreatectomy versus 23% after open distal pancreatectomy (P = 0.07) (8). Comparisons have been made between sutured and stapled control over the pancreatic stump, and the latter has proved to be the preferred option (6). Distal pancreatectomy can be undertaken not only by the preservation of the splenic vessels, namely, the Kimura technique (9), but also by the preservation of the short gastric vessels and the left gastroepiploic arcade, thereby enabling ligation of the splenic vessels, namely, the Warshaw technique (10, 11).

For higher-risk patients that include elderly, obese, and those with cancers, particularly, the pancreatic fistula rates have not shown to decrease sufficiently, whereby recommendations can be made as to what exact technique one should adopt to try and to reduce this almost inevitable risk. Sealants and other haemostatic agents have also been tried but remain largely experimental (6).

Importantly, the above studies demonstrate that regardless of whether the procedure is undertaken *via* a minimal access or open technique, the transection of the pancreas with the minimal risk of a pancreatic leak and bleeding is the ultimate goal.

This study reports the use of a new endovascular stapler made by Lexington Medical, Inc. (Bedford, MA, USA) called AEON™, which has a smoother ratchet and an increased angulation of the stapling device head as compared to the previously used stapler and how it may have affected the outcome in its use in the transection of the pancreas, with a reduction in the pancreatic fistula rates.

## Methods

Patients were selected from the Greater Manchester and Cheshire Cancer Network for hepatopancreatobiliary surgery and had all been through a multidisciplinary team meeting where they were assigned to undergo either a lateral or distal pancreatectomy for either a suspected or confirmed cancer or a benign pathology.

The hypothesis of the study was vetted through NHS online ethics decision tool and did not need any ethics approval (https://hra-decisiontools.org.uk/ethics/).

Patients underwent surgery in the standard lithotomy position with the main operator standing between the legs (although in a few cases, surgeon preference dictated a supine with a right tilt position). Access was obtained *via* an infraumbilical open technique using a 1-mm port (Kii port, Applied Medical™ California, USA). A further 12-mm port was placed cephalad and to the left of the initial camera port under direct vision with two 5-mm ports cephalad and to the right of the umbilicus as well as one to the far left, which also facilitated for drain placement in the lesser sac at the end of the operation with the minimum drain size used being 20 French gauge. In some cases, a Nathanson’s retractor (Cookmedical®, Ireland) was used with a small epigastric incision to hold the stomach in a cephalad direction once the lesser sac had been entered. In other cases, a simple suture through the stomach enables, again, cephalad retraction.

Dissection was performed with identification of the lesser sac and elevation of the stomach followed by creating a retro-pancreatic tunnel over the main portal vein after identification of the tumor in the pancreas to be removed. This maneuver again was undertaken as necessary depending on the location of the pancreatic tumor; so, for tail of pancreas lesions, it was not necessary for the pancreas body to mobilize off the underlying vessels more laterally. Mesenteric and omental dissection as well as ligation were carried out in most cases using the Lotus energy device (dissecting shears) (BOWA- electronic GmbH, Gomaringen, Germany). Open cases were carried out with the patient in the supine position with a left upper quadrant incision used, which was converted to a bilateral subcostal (rooftop) when required.

The pancreatic parenchyma was transected in all cases after a careful visualization of the left gastric and common hepatic artery using either a traditional vascular stapler or, more recently, the newly introduced AEON™ (Lexington Medical, Boston, MA, USA) vascular stapler using the “orange” cartridge. AEON™ stapler use commenced in early 2021. Both the 45- and 60-mm-length cartridges were available (normal range of 10 to 16 mm). However, in all cases, a 60-mm orange cartridge was used. Other methods used for transection of the pancreas, such as with ultrasonic energy devices and bipolar diathermy (n = 6), were excluded from the final analysis.

The AEON™ stapler utilizes a three-layer staple technology, like all staplers, but its staple lengths are uniform, and this is a unique difference of AEON™ compared with the other commonly used staplers, which have a graduated staple height, as well as its multi-firing gear to accommodate the thicker tissues (see **illustration**). In addition, the increased smoother articulation was subjectively observed, with no bleeding seen along the staple line in any case, similar to a randomized controlled trial in obesity surgery, with the conclusion that the AEON™ stapler causes less bleeding as a result of the staple technology used, but this cannot be ratified without a controlled study (12).

**Illustration:**
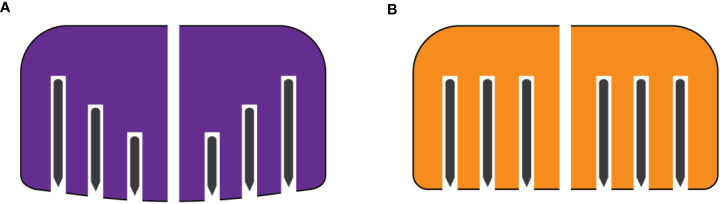
Other stapler depicting differing or graduated staple lengths **(A)**. AEON staple technology exhibiting uniform staple length **(B)**.

All patients went to a high dependency unit postoperatively with fluid sent from the drain on day 3 to measure for lipase (U/L) (normal range of 10 to 140 U/L). This study reports short-term outcomes, and so we have included data up to the first outpatient appointment after discharge, which is usually 1 month.

Demographic data (age and gender), details about patients’ comorbidities and disease profile, and data reflecting the postoperative outcome [length of stay, return to theatre rate, morbidity summarized by using comprehensive complication index (CCI) based on Clavien–Dindo classification (13), and mortality] were collected and recorded as per the Trust’s guidelines prospectively. The unit has regimented consultant lead ward rounds with good communication between colleagues and virtually harmonious surgical practices. CCI is recognized as a tool for reporting the cumulative burden of postoperative complications on a continuous scale, so it is recognized that it may differ when compared with one single complication.

Distal pancreatectomy fistula risk score (D-FRS) was calculated for all patients and compared between the AEON and the other stapler group to evaluate the cohort for selection bias. The data collection proforma was stored on a secure dataset by data managers. Outcome data of patients having their pancreas transected with AEON stapler were compared with the outcomes when other stapler was utilized. The inclusion and exclusion criteria of the patients that were analyzed for this study are listed as follows.

Inclusion criteria

- All consecutive patients having undergone distal pancreatectomy (open or minimally invasive) between January 2019 and November 2021;
- all indications (malignant and benign lesions) were included;
- patients requiring additional procedures (splenectomy, gastrectomy, adrenalectomy, etc.) for oncological clearance were also included.

Exclusion criteria

- Abandoned cases due to locally advanced or metastatic disease;
- patients not assessed for POPF as per the ISGPS (International Study Group of Pancreatic Fistula Group) (14) guidance

### Statistical analysis

Statistical analysis was performed using SPSS software (IBM Corp; IBM SPSS Statistics for Windows, version 23.0, Armonk, NY, USA). Categorical variables were compared using the chi-square test or Fisher exact test and numerical variables using one-way ANOVA test provided that the data had normal distribution, which was checked by the Kolmogorov–Smirnov test. Statistical significance was defined as <0.05.

## Results

### Patients’ profile

Fifty-eight patients underwent distal pancreatectomy between May 2019 and November 2021 (30 months) and comprise the study population. The median age was 63.5 years (range of 21 to 82). Both genders were represented equally in the sample (31 men and 27 women). The final diagnoses for the patients that underwent surgery were pancreatic ductal adenocarcinoma (24%), neuroendocrine tumors (29%), mucinous cystic neoplasms (23%), intraductal papillary mucinous neoplasms (5%), solid pseudopapillary tumors (10%), and metastatic lesions (9%). The proportion of various diagnoses/pancreatic pathology was not significantly different between the two groups. The same applies to patients’ comorbidities, the incidence of which was similar in both groups. No patients were excluded from the analysis as per the exclusion criteria as defined above, with all patients routinely undergoing drain lipase measurements on day 3. Demographics and disease profile are summarized in **Table 1**.

**Table 1:** Patients’ demographics and disease profile.

		AEON	Other staplers	P-value	Total
**Age (median, range)**		45 (25–80)	65 (21–82)	0.972	63.5 (21–82)
**Gender (M/F)**		8/7	23/20	1.000	31/27
BMI (n, %)					
** <30**		13 (87%)	39 (90%)	0.879	52 (90%)
** >30**		2 (13%)	4 (10%)		6 (10%)
**Ischaemic heart disease (n, %)**		0/15 (0%)	6/43 (14%)	0.107	6/58 (10%)
**Diabetes mellitus (n, %)**		3/15 (20%)	12/43 (28%)	0.562	15/58 (25%)
**Treatment with corticosteroids (n, %)**		0/15 (0%)	1/43 (2%)	1.000	1/58 (1.7%)
**Treatment with anticoagulants (n, %)**		2/15 (13%)	3/43 (7%)	0.456	5/58 (9%)
Diagnosis (n, %)					
PDAC		1 (7%)	13 (30%)	0.156	14 (24%)
PNET		3 (20%)	14 (33%)		17 (29%)
MCN		5 (33%)	8 (19%)		13 (23%)
IPMN		2 (13%)	1 (2%)		3 (5%)
Solid		3 (20%)	3 (7%)		6 (10%)
pseudopapillary tumors Metastases		1 (7%)	4 (9%)		5 (9%)
Surgery (n, %)					
Open		5 (33%)	11 (26%)	0.874	16 (28%)
Laparoscopic		8 (53%)	32 (74%)		40 (69%)
Robotic		2 (14%)	0		2 (3%)
**D-FRS (mean ± SD)**		31.6 ± 18.2	34.4 ± 18.2	0.644	n/a

D- FRS, distal pancreatectomy fistula risk score (https://www.evidencio.com/models/show/2573).

### Perioperative management and outcomes

Distal pancreatectomy was the main procedure for the entire study population. Open approach was followed in 16 cases (28%), laparoscopic in 40 (69%), and robotic in 2 (3%). Thirty-four procedures (59%) were combined with splenectomy, and the remaining 24 (41%) were spleen-preserving distal pancreatectomies. Simultaneous local liver resection (for an insulinoma, small superficial metastases in segment III), gastrectomy, adrenalectomy, or cholecystectomy was performed in 11 cases. For the division of the pancreas, Lexington AEON stapler with the orange cartridge was utilized in 15 cases (26%) and endo-GIA vascular or linear staplers in 43 cases (64%). Pancreatic fistula was diagnosed postoperatively in 31 patients (53%) (32 patients with biochemical leak and one with grade C POPF). Overall morbidity was 60%, and the mean CCI was 11.31. One patient returned to theatre because of postoperative haemorrhage. Ninety-day mortality was 1.7%. The 2% death was in the “other” stapler group and unrelated directly to a complication from the transection of the pancreas, but the patient did develop sepsis secondary to a pancreatic leak. The study outcomes are presented in detail in **Table 2**.

**Table 2:** Postoperative outcome comparison between AEON and other instrument used for pancreatic transection.

	AEON	Other staplers	P-value
**Drain lipase (mean ± SD)**	446 ± 878 U/L	4.208 ± 5717 U/L	0.018
**POPF (n, %)**	3/15 (20%)	28/43 (65%)	0.001
** Grade A**	3	27	
** Grade B**	0	0	
** Grade C**	0	1	
**Comprehensive complication index (mean ± SD)**	4.97 ± 6.88	13.80 ± 18.19	0.087
**Length of stay (LOS) (mean ± SD)**	6.2 ± 2.51	8.74 ± 3.72	0.018
**Mortality (n, %)**	0/15 (0%)	1/43 (2%)	1.000

Incidence of pancreatic fistula (p < 0.001) and LOS (p<0.018) were significantly reduced with the use of the AEON stapler. The CCI was also notable better although this did not reach statistical significance. POPF recorded as per ISGPS 2016 updated guidelines, ref. 12.

A = biochemical leak. B = requires a change in the postoperative management; drains are either left in place >3 weeks or repositioned through endoscopic or percutaneous procedures. C = postoperative pancreatic fistula refers to those postoperative pancreatic fistulas that require reoperation or lead to single or multiple organ failure and/or mortality attributable to the pancreatic fistula.

### Lexington AEON stapler. A comparison with another stapler

**Figure 1:**
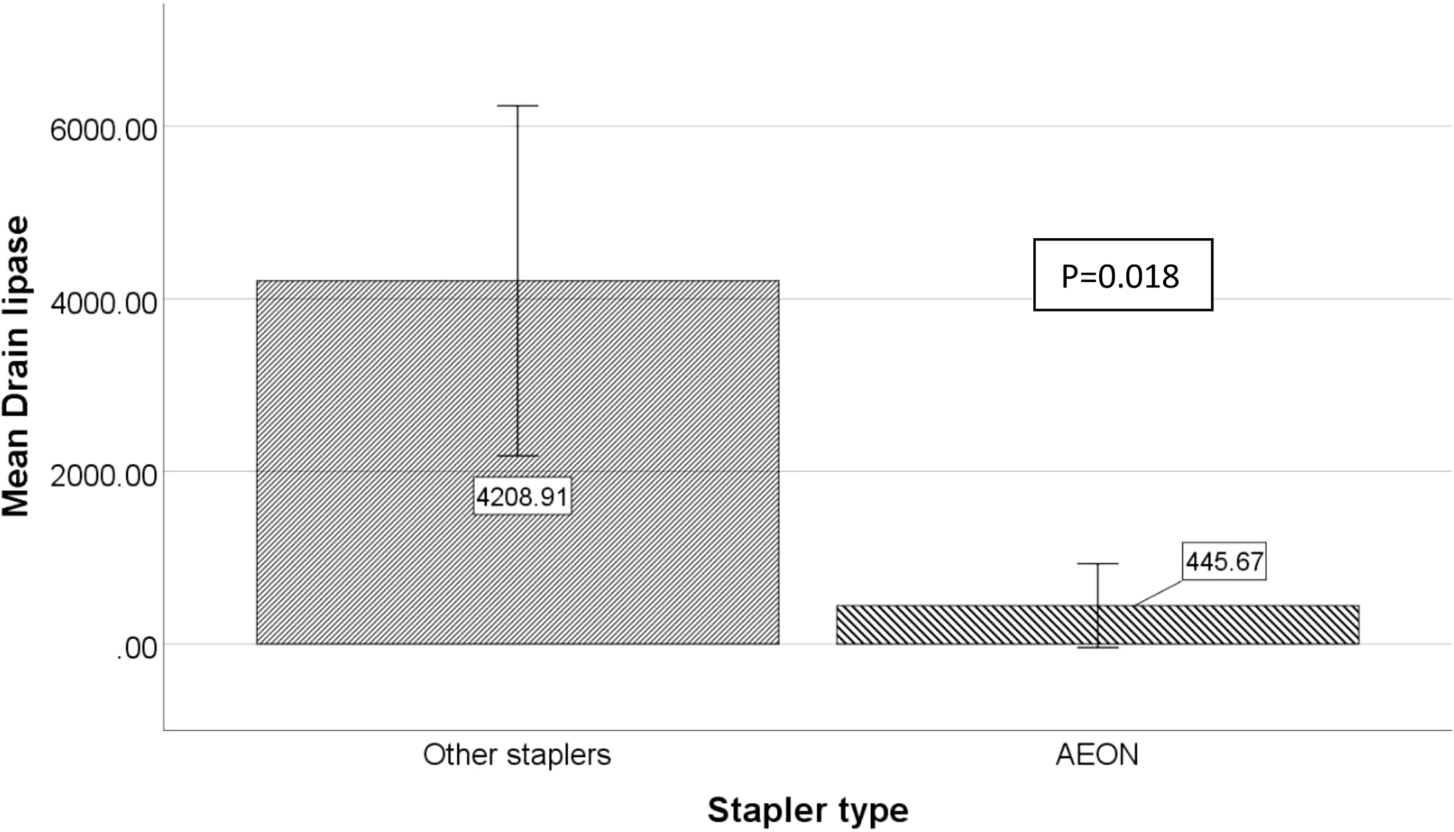
Comparison of mean drain lipase value, measured at postoperative day 3, between AEON and the other stapler used for pancreatic transection. Mean drain lipase at postoperative day 3 was significantly higher in patients having their pancreas divided with other instrument (4,209 U/L) versus AEON (446 U/L); 95% confidence intervals are shown.

**Figure 2:**
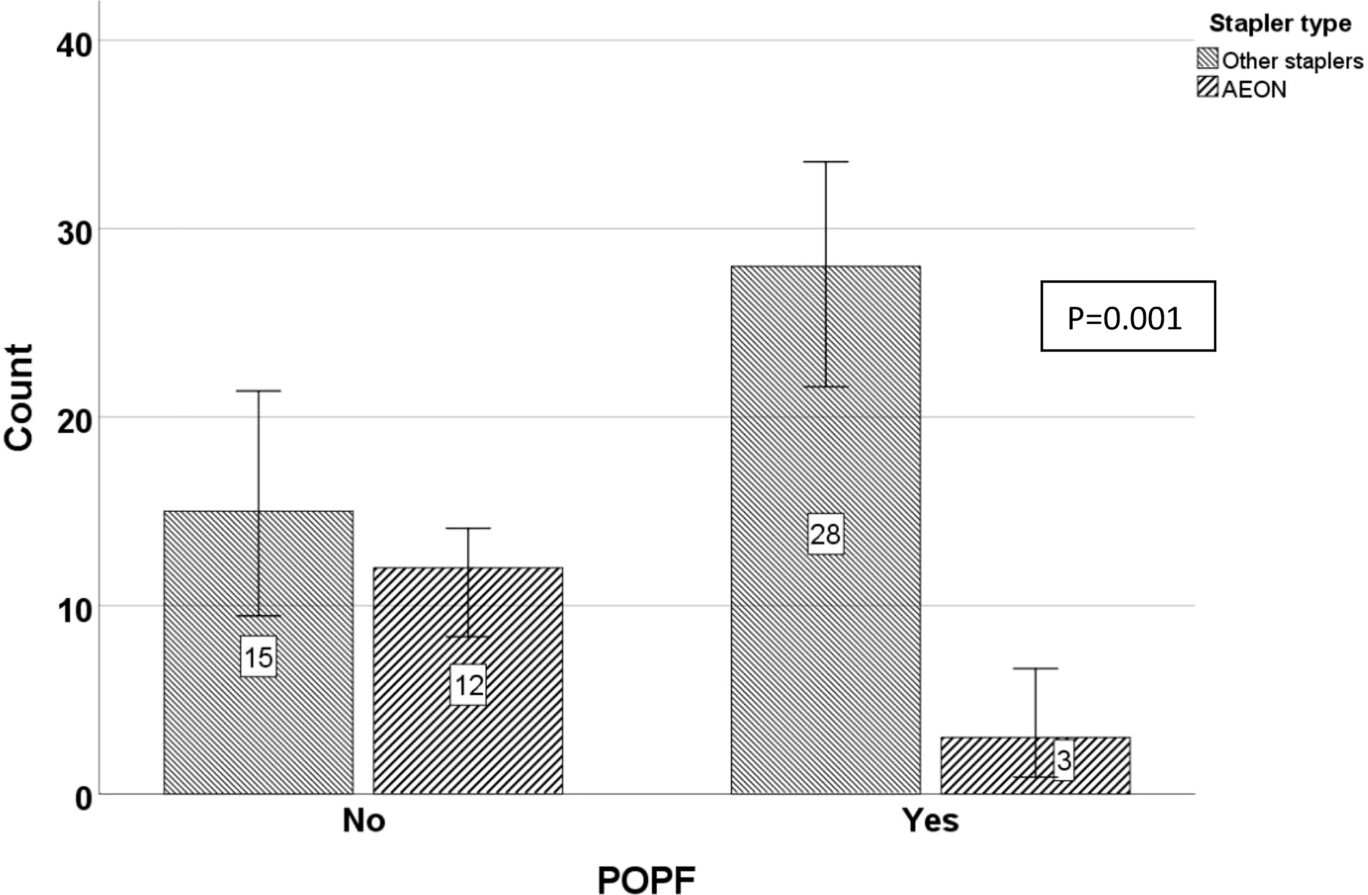
Comparison of postoperative pancreatic fistula (POPF) incidence between AEON and another stapler used for pancreatic transection. Using the AEON stapler, the pancreatic leak rate was significantly lower when using AEON stapler (p = 0.001). All in the AEON group were A; in another stapler, all were A, save one which was a C.

**Figure 3:**
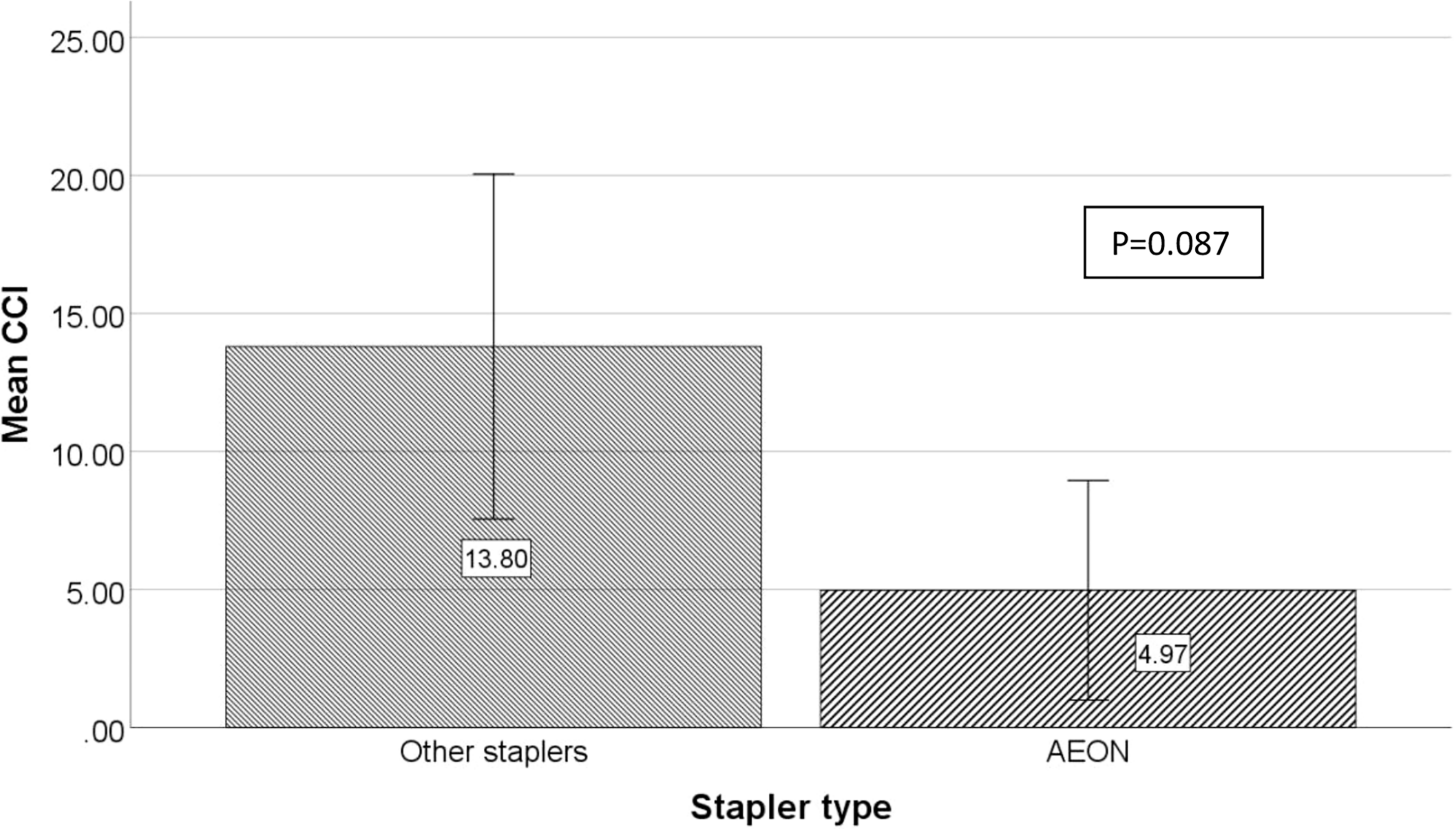
Comparison of mean comprehensive complication (CCI) index between AEON and other staplers used for pancreatic transection. The mean CCI when AEON was used was 4.97, increasing to 13.80 when other instrument were used (p = 0.087).

**Figure 4:**
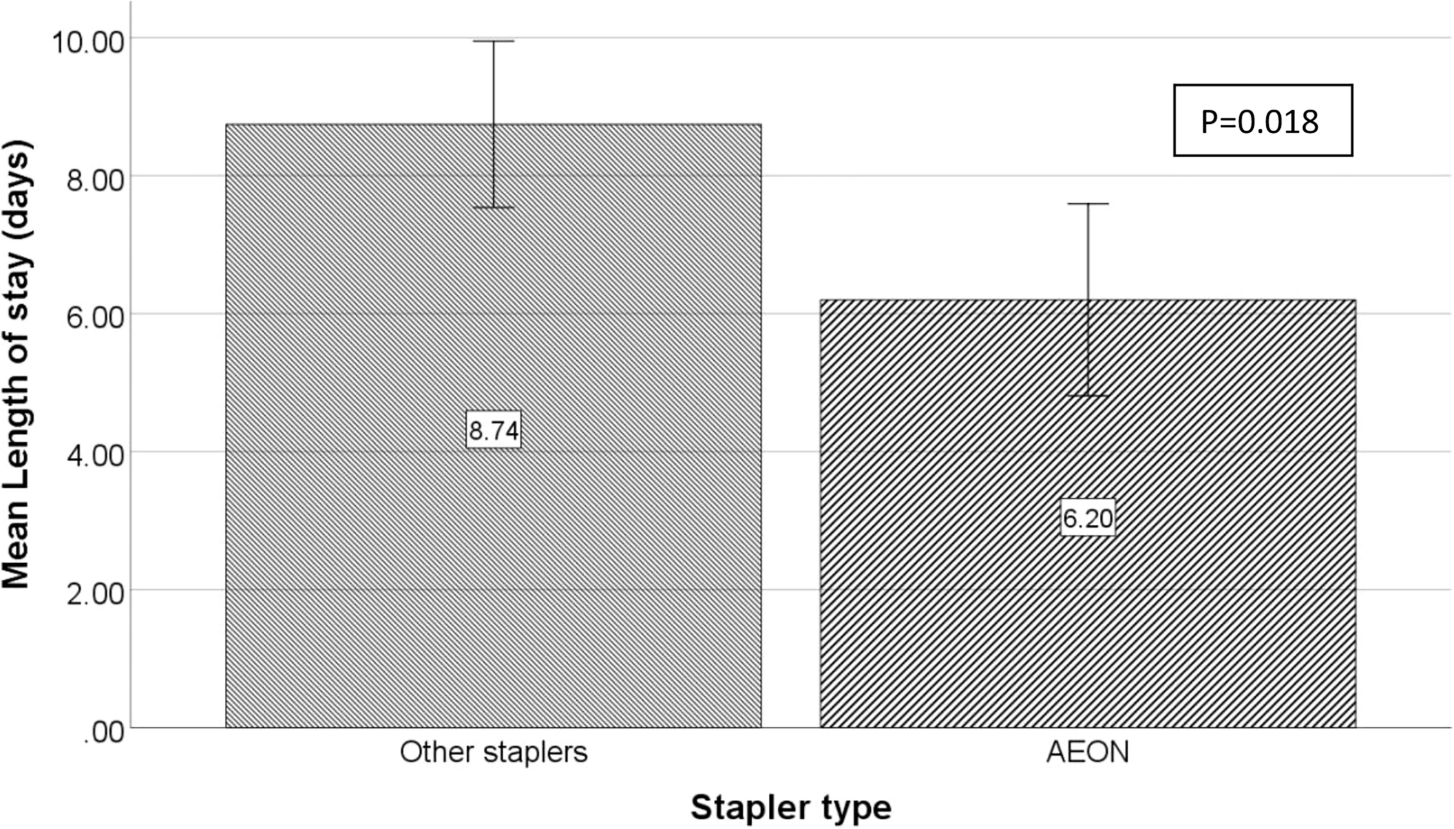
Comparison of mean length of stay between AEON and other staplers used for pancreatic transection. The mean length of stay was shorter by 4 days in the AEON group compared with that in the other instrument (6 and 9 days, respectively; p = 0.018).

Mean drain lipase at postoperative day 3 was significantly higher in patients having their pancreas divided with other staplers (4,208 U/L) compared with that with AEON (446 U/L) (**Figure 1**). The difference was statistically significant (p = 0.018). Expectedly, this result reflects the pancreatic leak rate, which again was significantly lower when using AEON stapler (65% reduced to 20%) (p = 0.001) (**Figure 2**). In terms of postoperative complications, the mean CCI when AEON was used was 4.97, increasing to 13.80 when the other stapler was used (p = 0.087) (**Figure 3**). This was not significant as compared to the leak rate. The mean length of stay was shorter by 3 days in the AEON group compared with that in the other instrument (6 and 9 days, respectively; p = 0.018) (**Figure 4**). D-FRS was almost similar in both groups (31.6 in AEON versus 34.4 in the other stapler group, p = 0.644). **Table 2** summarizes the comparison of outcomes between the two groups.

## Discussion

In this brief overview of a very early experience using the AEON™ stapler, there is a clear demonstration, from the results, of a significant reduction in pancreatic fistula rates from 65% to 20% (p = 0.017), which was relevant especially as the D-FRS demonstrated no selection bias. Subjectively, a noted reduction in drain fluid output after the AEON™ stapler was introduced into the surgical armamentarium in the unit, leading to an investigation to perform an early evaluation of any difference in the pancreatic leak rates in a more scientific and objective manner. The unit already had a low Clavien–Dindo B and C rates with return to theatre as a rare event as reflected in **Table 2**, mainly due to the regimental surgical practices, with a drain being the standard of care as well as follow-up protocols with the emphasis on a consultant lead service. This study also demonstrated scientifically that the reduced pancreatic leak rate had an expected effect with the noted reduction in CCI index from just over 13 to just under 5. Laparoscopic distal pancreatectomy remained the preferred method of surgery, which is confirmed by various reviews and systematic analysis showing that the benefits of the laparoscopic approach, mainly with reduced hospital stay but, disappointingly, with no significant difference in the fistula rates were demonstrated with minimal access surgery (13), which underlines the relative importance of continued emphasis in reducing the POPF by possibly the introduction of more innovative techniques.

The results from the data of this study are comparable with that of other large cohort studies in terms of spleen preservation and indications for surgery; although the majority of the cases were laparoscopic, open techniques were also included in the study as the comparison was on the pancreatic fistula rate based on the transection technique used (4). However, one issue that was of concern was a very high fistula rate in the unit of over 70% albeit mainly A, which is now reduced to be in line with the ISGPS guidelines, where studies have shown rates of between 16.8% and 21.7% in distal pancreatectomy (14, 15) (see **Table 1**). POPF rate remains the main contentious issue for pancreatic surgeons and will continue to be debated for years to come especially in terms of what is the presumed best method to reduce or even, altogether, avoid a leak from the transected pancreatic stump. The ideal transection technique could be regarded as a major determinant of a reduced leak rate, which will inevitably have a net positive effect with other factors such as a decreased length of stay and the overall morbidity. This driving measure of success of a distal pancreatectomy has created much controversy in the literature with comparisons of suture versus stapled methods to control fistula rates as shown in the meta-analysis of Wang et al. (16), two high-volume institutional studies (17, 18) and two RCT (19, 20), which all found that stapled stump closure was associated with a slightly higher POPF rate. One large study from Boston, USA, demonstrated examining 14 years of 462 consecutive distal pancreatectomies with an overall fistula rate of 29% (21), which is greater than what this study experienced with 21.4% using the AEON™ stapler. The study by Ferrone et al. (20) compared the various techniques including suture closure, falciform patch, as well as staple line closure with and without a reinforcement, with the leak rates ranging from 24% to 33%, but no significant difference was found regardless of the technique used. Importantly, the multivarious analysis found that BMI > 30 kg/m^2^, male gender, and an additional procedure undertaken were significant predictors of a pancreatic fistula. The AEON™ stapler utilizes technology not apparent in other staplers with a uniform staple line height (see illustration) with the orange staple cartridge and gear adjustment for use in thicker tissue (12). One study reported a higher fistula rate in patients with a thicker pancreas (>12mm) (22), with other studies recommending a gradual graded approach to pancreatic transection, lasting 2 to 3 min as this may reduce the POPF rate (23, 24). The data in this study are not robust enough to comment on the various techniques that were used in terms of whether a graded technique was preformed or not, but it can comment on the D-FRS being similar in the groups analyzed depicting no selection bias in the cases analyzed. In addition, there is an accepted recognition that normal pancreatic tissue adjacent to a tumor in the pancreas is normally thickened, with the technology used having a longer and more uniform staple line, which possibly was most likely responsible for the dramatic reduction in the pancreatic fistula rate from 65 to 20%. As there was no single surgeon involved in the cases presented using either stapler, but instead at least six surgeons utilizing both staplers, surgical technique bias is unlikely to have influenced the result. The reduction in CCI (p = 0.087) was no quite significant, which is somewhat unexpected as the fistula rate was significantly reduced (p = 0.018), but CCI is recognized as a tool for reporting the cumulative burden of postoperative complications on a continuous scale, so it is entirely reasonable that it may differ from when comparing with one single complication.

Finally, there were no reported returns to theatre for bleeding in either group, so both devices used are considered safe and effective to use, with one although leaning towards a significant advantage in reduction of pancreatic fistula rates.

The limitation of this study remains that it is a retrospective review based on a rather small cohort of patients. However, the clinical recognition by the unit of a notable difference in the drain output and the drain lipase measurements after switching to the AEON™ stapler was considered important to investigate and report especially as a reduction in the POPF rate is probably the single most important postoperative outcome measurement for distal or lateral pancreatectomy. It would be very reasonable now to determine the validity of the stapler in the near future with a further follow-up study with greater numbers or even a randomized trial to provide higher level of evidence.

In summary, despite the small numbers, the authors report a genuine result, and it seems very unlikely that they were by chance.

Further evaluation from other centers based on larger cohorts is already being planned to ratify the results of this preliminary evaluation, but these data can recommend that the AEON™ stapler with the orange cartridge plays a positive role in the transection of the pancreas with a comparable if not improved fistula rate.

## Data availability statement

The original contributions presented in the study are included in the article/supplementary material. Further inquiries can be directed to the corresponding author.

## Author contributions

All authors contributed to the article and approved the submitted version.
